# Novel lineages of bacteria with reduced genomes from the gut of farm animals

**DOI:** 10.1128/msphere.00294-25

**Published:** 2025-06-30

**Authors:** Shahjahon Begmatov, Alexey V. Beletsky, Andrey V. Mardanov, Anastasia P. Lukina, Liubov B. Glukhova, Olga V. Karnachuk, Nikolai V. Ravin

**Affiliations:** 1 Institute of Bioengineering, Research Center of Biotechnology of the Russian Academy of Sciences, Moscow, Russia; 2 Tomsk State University, Tomsk, Russia; University of Wisconsin-Madison, Madison, Wisconsin, USA

**Keywords:** gut microbiome, reduced genome, evolution

## Abstract

Genome reduction and associated metabolic deficiencies have been described in various lineages of parasitic and symbiotic microorganisms that obtain essential nutrients from their partners, and in some free-living microorganisms that inhabit stable environments. The animal gut is a relatively stable ecosystem, characterized by an abundance of organic substances and a high concentration of microorganisms, which provides favorable conditions for the survival of microorganisms with reduced genomes. Metagenomic analysis of 49 samples of feces of farm animals (cows, sheep, yaks, and horses) revealed uncultured lineages of bacteria with reduced genomes (<1 Mbp): family UBA1242 (*Christensenellales*, *Firmicutes*), order Rs-D84 (*Alphaproteobacteria*), and family UBA9783 (*Opitutales*, *Verrucomicrobiota*), defined in genome-taxonomy database. Analysis of the genomes showed that these bacteria lacked pathways for the biosynthesis of amino acids, nucleotides, lipids, and many other essential metabolites. The UBA9783 genomes encoded a near-complete Embden-Meyerhof glycolytic pathway and the non-oxidative phase of the pentose phosphate pathway, while in UBA1242 and Rs-D84, these pathways are incomplete. All bacteria are limited to fermentative metabolism and lack aerobic and anaerobic respiratory pathways. All UBA9783 and some Rs-D84 genomes encoded F_0_F_1_-type ATP synthase and pyrophosphate-energized proton pump; they also can import and utilize peptides and some amino acids. While UBA9783 bacteria could thrive as specialized free-living organisms in the organic-rich gut environment, the UBA1242 and Rs-D84 lineages appear to have adopted the lifestyle of an obligate symbiont/parasite, obtaining metabolites from other cells.

**IMPORTANCE:**

The microbiota of the animal gastrointestinal tracts is a complex community of microorganisms which interact in a synergistic or antagonistic relationship and play key nutritional and metabolic roles. However, despite its importance, the gut microbiota of farm animals, especially its uncultured majority, remains largely unexplored. We performed a metagenomic analysis of the gut microbiome of farm animals and characterized three uncultured lineages of bacteria with reduced genomes (<1 Mbp) from the phyla *Firmicutes*, *Proteobacteria*, and *Verrucomicrobiota*. These bacteria were predicted to possess key metabolic deficiencies such as the inability to synthesize essential cell metabolites, suggesting their adaptation to the lifestyle of a symbiont/parasite, or a scavenger obtaining nutrients from the organic-rich gut environment. This study shows that genome reduction with metabolic specialization and adaptation to a partner-dependent lifestyle occurred through convergent evolution in several phylogenetically distant lineages of gut microbiota.

## OBSERVATION

Bacterial genome sizes range from <0.5 to >10 Mbp. Bacteria with large genomes typically have diverse metabolic capabilities and can adapt to changing environmental conditions. Genome reduction, which is thought to reduce the metabolic burden for basic cellular processes (1), is observed primarily in two cases. The first example is symbiotic or parasitic organisms that receive the necessary metabolites from their partners. Extreme cases of genome reduction have been described in intracellular bacteria of insects whose genomes are reduced to 139–250 kbp (1). The second case is microorganisms with a specialized metabolism inhabiting stable ecosystems, such as the marine bacteria *Pelagibacter* and *Prochlorococcus* (2).

The animal intestine is a relatively stable ecosystem, characterized by an abundance of organic substances and a high concentration of microorganisms (up to 10^10^ cells/mL) (3), which provides favorable conditions for the survival of microorganisms with reduced genomes, both partner-dependent and free-living. Because such organisms have various metabolic deficiencies, they mainly represent uncultured lineages that were identified through metagenomic studies. The existence of microorganisms with reduced genomes belonging to *Actinobacteriota*, *Firmicutes*, *Bacteroidota*, and *Proteobacteria* in the human intestinal microbiota has been reported (4). Particularly, an uncultivated order RF39 within the class *Bacilli* has a highly reduced genome and numerous auxotrophies (4). A number of near-complete metagenome-assembled genomes (MAGs) with sizes of <1 Mbp were obtained from the chicken gut (5), but their metabolic potential has not been reported.

The main objective of this investigation was to identify, characterize, and compare phylogenetically distant bacteria with reduced genomes living in the animal gut. We performed a metagenomic analysis of 49 samples of feces of farm animals (6 camels, 4 yaks, 20 cows, 5 horses, and 14 sheep). After collection, the samples were frozen and delivered to the laboratory where total DNA was extracted using the DNeasy Power Soil Pro Kit (Qiagen). For each sample, total DNA was sequenced on an Illumina NovaSeq 6000 platform as described earlier (6). Contigs were *de novo* assembled using MEGAHIT v.1.2.9 (7) and binned into MAGs using MetaBat2 v.2:2.15 (8). The completeness of MAGs and their redundancy (contamination) were assessed using CheckM2 (9). The assembled MAGs were taxonomically identified using the GTDB-Tk v.2.4.0 tool (10) according to the Genomic Taxonomy System (GTDB) (11). Genes were identified using Prodigal v.2.6.3 (12). Pangenome analysis was performed using METABOLIC (13) and Anvi’o v.8 with parameter –minbit 0.25 (14).

Of the 1,388 MAGs with >80% completeness and <10% contamination, 28 MAGs were within 1 Mbp in size. Four genomes belonged to *Firmicutes* (*Bacillota*) of the uncultured family UBA1242 (order *Christensenellales*), eight MAGs were assigned to the candidate genus *Enterousia* (uncultured order Rs-D84 of *Alphaproteobacteria*), and one represented the uncultured family UBA9783 of the order *Opitutales*, phylum *Verrucomicrobiota*. One obtained *Enterousia* MAG with a size just above the 1 Mbp threshold was added to the data set. The sizes of these MAGs ranged from 747.6 to 1,015.1 kbp (Table 1). Inspection of GTDB showed that the vast majority of other members of UBA1242, *Ca*. Enterousia, and UBA9783 also have genomes smaller than 1 Mbp. Moreover, genome reduction is a common property of all representatives of the Rs-D84 order. Since these uncultured bacterial lineages had not been previously characterized, we analyzed their metabolic potential. The objects for comparative analysis and metabolic reconstructions, in addition to the obtained MAGs, were 40 genomes of the family UBA1242, 10 genomes of the order Rs-D84, and 3 genomes of the family UBA9783, representing different genera. The vast majority of these genomes were obtained from animal gut-related sources (feces, rumen, etc.).

**TABLE 1:** Main properties of the genomes

MAG ID	Completeness/contamination (%)	Contigs	Contig N50 (bp)	Coding density	Genome size (bp)	Genes	Taxonomy (genus)^*a*^	GenBank
*Firmicutes*; *Clostridia*; *Christensenellales*; f_UBA1242								
KG228-83	88.3/0.37	97	11,647	0.928	762,131	738	HGM11372	JBKSMV000000000
KA0021_177	80.12/0.15	168	6,590	0.903	921,973	930	Onthoplasma	JBKSMT000000000
KY236-132	89.78/0.23	58	20,294	0.915	791,354	782	Onthoplasma	JBKSNA000000000
KA0036-72	89.29/0.67	135	8,034	0.911	894,143	868	UBA5489	JBKSMU000000000
*Proteobacteria*; *Alphaproteobacteria*; o_Rs-D84; f_Rs-D84								
101_22-10	94.2/0.73	32	50,859	0.902	897,596	904	Enterousia	JBKSNC000000000
103_22-226	90.83/7.35	221	5,932	0.902	1,015,128	1195	*Enterousia*	JBKSMQ000000000
125_22-69	93.74/0.22	68	18,084	0.906	872,604	901	*Enterousia*	JBKSMR000000000
82_22-119	93.75/0	21	73,863	0.900	891,946	887	*Enterousia*	JBKSMS000000000
KH370_54	87.93/0.72	135	7,707	0.914	747,628	820	*Enterousia*	JBKSNB000000000
KH383_101	96.2/2.38	84	14,052	0.914	786,106	827	*Enterousia*	JBKSMW000000000
KS320-82	93.71/0.1	45	30,663	0.904	876,159	891	*Enterousia*	JBKSMY000000000
KS318-84	96.38/0	40	26,548	0.892	849,589	844	*Enterousia*	JBKSMX000000000
KS323-146	91.9/4.33	212	4,559	0.905	810,804	957	*Enterousia*	JBKSMZ000000000
*Verrucomicrobiota*; *Verrucomicrobiae*; *Opitutales*; f_UBA9783								
101_22-192	95.98/0	28	67,407	0.91	984,270	940	Unclassified	JBKSMP000000000

^*a*^ According to GTDB Release 220.

The genome sizes ranged from 671 to 1,611 kbp, and the fraction of coding sequences was 81%–94%, which matches a typical range of 85%–90% for bacterial genomes and indicates the absence of pseudogene accumulation characteristic of obligate pathogens undergoing active genome decay (1).

UBA9783 and Rs-D84 bacteria have rod-shaped cells since their genomes encode the rod-shape determining proteins RodA and MreB, as well as the peptidoglycan D,D-transpeptidase MrdA. Flagellar machinery was missing in all three lineages, but UBA9783 genomes contained genes for type IV pili enabling twitching motility. The finding of sporulation genes in the UBA1242 family was quite unexpected, since spore formation is usually lost in the reduced-genome lineages within the *Firmicutes* (15).

**Fig 1:**
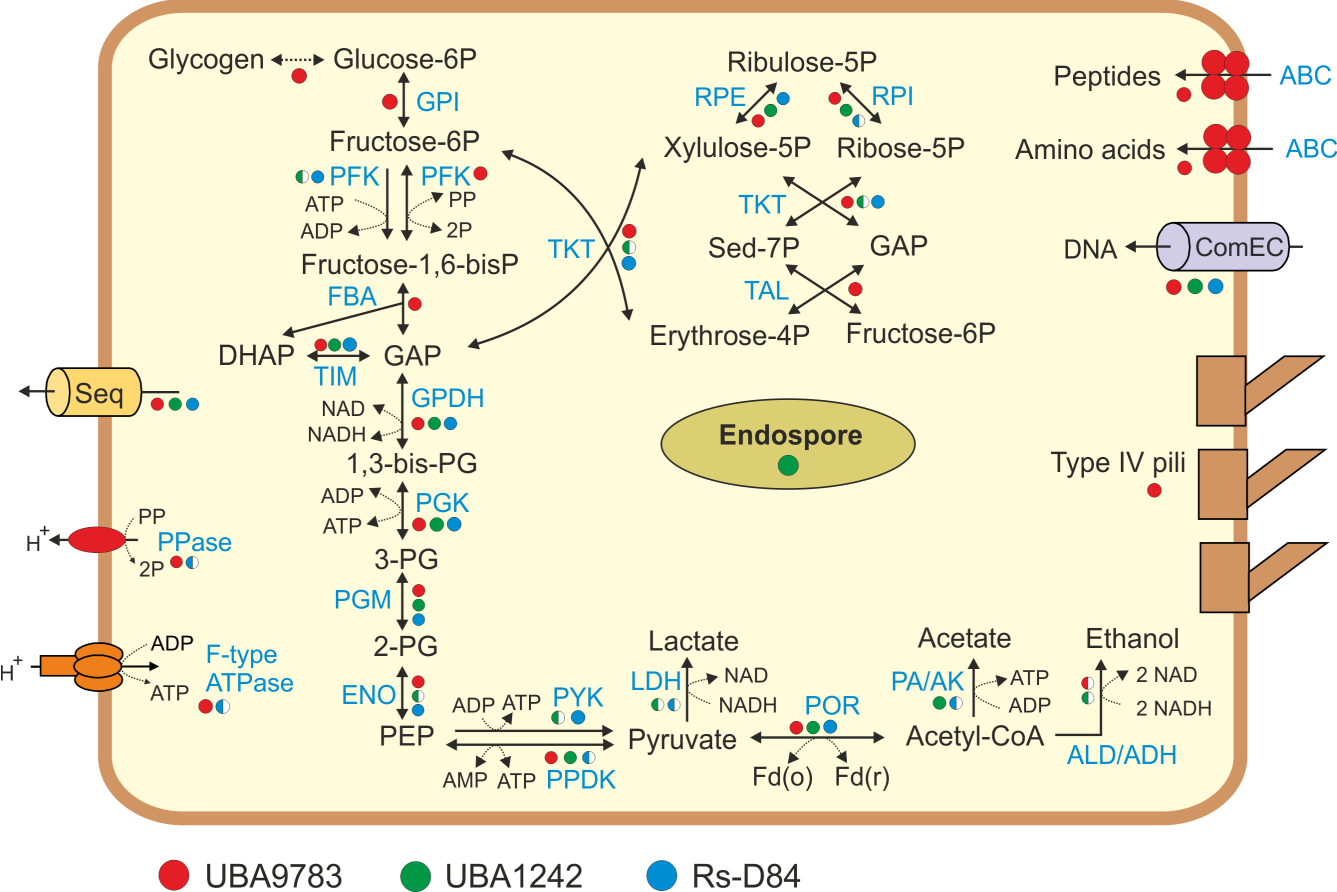
Overview of the main metabolic pathways. Enzymes: GPI, glucose-6-phosphate isomerase; PFK, phosphofructokinase; FBA, fructose-bisphosphate aldolase; TIM, triose phosphate isomerase; GPDH, glyceraldehyde 3-phosphate dehydrogenase; PGK, phosphoglycerate kinase; PGM, phosphoglycerate mutase; ENO, enolase; PYK, pyruvate kinase; PPDK, pyruvate, phosphate dikinase; Rpe, ribulose-5-phosphate epimerase; Rpi, ribulose-5-phosphate isomerase; Tal, transaldolase; Tkt, transketolase; POR, pyruvate ferredoxin oxidoreductase; LDH, lactate dehydrogenase; PA/AK, phosphate acetyltransferase and acetate kinase; ALD, aldehyde dehydrogenase; ADH, alcohol dehydrogenase; PPase, pyrophosphatase; ABC, ABC-type transporters. Other abbreviations: GAP, glyceraldehyde-3-phosphate; PG, phosphoglycerate; PEP, phosphoenolpyruvate; Sed-7P, sedoheptulose-7-phosphate; PP, pyrophosphate; P, phosphate. The presence of enzymes is indicated by circles: filled (in >80% of genomes), and half-filled (in <80% but in at least one of genomes).

The UBA9783 genomes encoded a near-complete Embden-Meyerhof glycolytic pathway from glucose-6-phosphate to pyruvate and the non-oxidative stage of the pentose phosphate pathway (Fig. 1). Pyruvate kinase was not found, but interconversion of pyruvate and phosphoenolpyruvate could be performed by pyruvate, phosphate dikinase. Pyrophosphate-dependent phosphofructokinase, enabling reversible conversion of fructose-6-phosphate into fructose 1,6-bisphosphate, was encoded instead of the classical ATP-dependent enzyme. Therefore, all steps of glycolysis and gluconeogenesis could be performed. Pyruvate: ferredoxin oxidoreductase could catalyze the interconversion of pyruvate and acetyl-CoA. The latter can be converted to ethanol via an acetaldehyde intermediate by the bifunctional acetaldehyde-CoA/alcohol dehydrogenase present in some UBA9783 genomes, while enzymes enabling the production of acetate, lactate, or formate have not been found. The tricarboxylic acid cycle is incomplete (only succinyl-CoA ligase and fumarate hydratase were found) and is probably used for biosynthetic purposes. Genome analysis showed the absence of the respiratory chain (complexes I–IV), as well as known reductases for aerobic and anaerobic respiration. The genomes encoded F_0_F_1_-type ATP synthase, which can use the transmembrane proton gradient for ATP production. This finding was unexpected because the only found primary proton pump capable of generating such a gradient was membrane-bound pyrophosphatase. Complete pathways for the biosynthesis of amino acids, nucleotides, and fatty acids were missing, and these compounds should be obtained from other organisms. Genes for secreted glycoside hydrolases and proteolytic enzymes, as well as sugar transporters, were not found. On the contrary, UBA9783 genomes encoded ABC-type transporters for the uptake of oligopeptides and amino acids, which probably could be used as substrates. In addition, genes encoding competence proteins enabling the uptake of DNA from the environment were identified.

Although Rs-D84 and UBA1242 are similar in genome size to UBA9783, their metabolic potential is more limited. Their genomes lacked glucokinase, glucose-6-phosphate isomerase, and fructose-bisphosphate aldolase, and about half of the UBA1242 genomes also lacked the enolase gene. These bacteria, therefore, should obtain glycolytic intermediates and/or pyruvate from the environment or from host organisms, as suggested for *Patescibacteria* (16). Some genomes contained genes for phosphate acetyltransferase and acetate kinase, which enable the oxidation of acetyl-CoA to acetate with concomitant synthesis of ATP, as well as genes for acetaldehyde dehydrogenase, alcohol dehydrogenase, or lactate dehydrogenase. Most genomes contained genes for the non-oxidative step of the pentose phosphate pathway, with the exception of transaldolase. The tricarboxylic acid cycle and the respiratory chains are absent, which is consistent with previous data on *Enterousia* genomes (17). The F_0_F_1_-type ATP synthase and pyrophosphate-energized ion pump were found in some Rs-D84 genomes but were missing in UBA1242. The Rs-D84 and UBA1242 genomes lacked standard transporters for the uptake of sugars, amino acids, and peptides, but like UBA9783 genomes, they encoded a variety of hypothetical membrane-bound proteins that may be involved in transport and/or interactions with other cells. It is possible that these bacteria may acquire metabolites from a partner organism via intercellular connections, as suggested for *Patescibacteria* (16).

The results show that three uncultured, phylogenetically distant bacterial lineages from the animal gut, families UBA9783 (*Verrucomicrobiota*), UBA1242 (*Firmicutes*), and order Rs-D84 (*Alphaproteobacteria*) have reduced genomes with limited metabolic capacities and may be free-living scavengers (UBA9783) or have a partner-dependent lifestyle. The observed metabolic deficiencies, such as the loss of biosynthetic pathways for key metabolites, the absence of respiratory chains, and some genes of glycolysis, are similar to those previously described for bacteria of the phylum *Patescibacteria*, which includes only obligate partner-dependent organisms.

## Data Availability

The genome sequences have been deposited in NCBI GenBank database within the BioProject PRJNA785979.
